# Prosociality in a despotic society

**DOI:** 10.1016/j.isci.2023.106587

**Published:** 2023-04-08

**Authors:** Debottam Bhattacharjee, Eythan Cousin, Lena S. Pflüger, Jorg J.M. Massen

**Affiliations:** 1 Animal Behavior & Cognition, Department of Biology, Utrecht University, Padualaan 8, 3584 CH Utrecht, the Netherlands; 2 Department of Ecology, Physiology & Ethology, Faculty of Life Sciences, University of Strasbourg, 67000 Strasbourg, France; 3 Department of Behavioral and Cognitive Biology, University of Vienna, Djerassiplatz 1, 1030 Vienna, Austria; 4 Austrian Research Center for Primatology, Ossiach 16, 9570 Ossiach, Austria

**Keywords:** Biological sciences, Zoology, Evolutionary biology

## Abstract

Prosociality is the intent to improve others’ well-being. Existing hypotheses postulate that enhanced social tolerance and inter-individual dependence may facilitate prosocial preferences, which may favor the evolution of altruism. While most studies are restricted to “tolerant” (cooperatively breeding and self-domesticated) species, despotic societies provide an alternative opportunity to investigate prosociality due to nepotism and ample inter-individual dependencies. Japanese macaques live in hierarchical matrilineal societies, with strong kin bonds. Besides, tolerance among non-kin may persist through reciprocity. Using a group service food-provision paradigm, we found prosocial preferences in a semi free-ranging group of Japanese macaques. The extent of provisioning was at levels comparable to tolerant species. Dyadic tolerance predicted the likelihood and magnitude of provisioning, while kinship predicted the magnitude. We emphasize the role of a complex socio-ecology fostering individual prosocial tendencies through kinship and tolerance. These findings necessitate a framework including different forms of interdependence beyond the generally tolerant species.

## Highlights

• Conventionally, research on prosociality is restricted to tolerant societies
• We show despotic semi free-ranging Japanese macaques have prosocial preferences too
• Dyadic social tolerance and kinship positively predict prosocial provisioning
• Selective prosocial preferences may be maintained by inter-individual dependencies

## Graphical abstract

**Figure:**
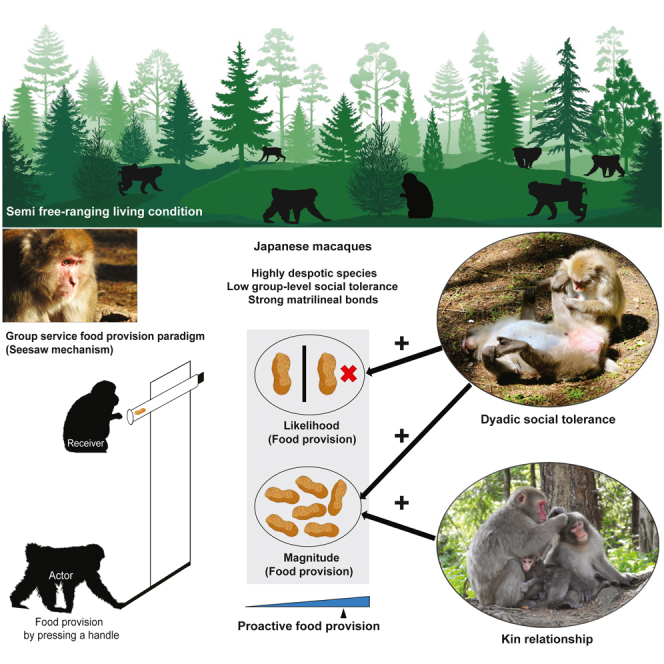

## Introduction

Prosociality is the tendency to help and enhance the welfare of others.^1^^,^^2^ Individuals who prefer outcomes that benefit others are considered to have prosocial preferences. We, humans, for instance, have generalized prosocial preferences that lead us to behave altruistically at an exceptionally high level, even to strangers without any potential for (direct) reciprocity.^3^^,^^4^ (also see^5^). In addition to “cognitively less-demanding” generalized reciprocity where individual recognition is not a pre-requisite, key mechanisms favoring prosocial preferences can be specific to certain individuals via partner choice which includes kin selection and direct reciprocity, and indirect reciprocity (in humans).^3^^,^^5^^,^^6^ Therefore, a clear distinction can be made between selective (through kinship and direct reciprocity) and unconditional prosocial preferences (generalized prosocial preferences).^7^ In search of the evolutionary trajectory of prosociality, comparative psychologists examined and found empirical evidence of prosocial preferences in a wide range of species.^8^^,^^9^ Two hypotheses, in particular, gained empirical support in explaining the evolution of prosociality. The self-domestication hypothesis considers prosociality a by-product of selection against reactive aggression, resulting in increased social tolerance.^10^^,^^11^ On the other hand, the cooperative breeding hypothesis specifically points out allomaternal care, in other words, considerable inter-individual dependence in a group,^12^ fostering prosociality.^8^^,^^10^^,^^13^^,^^14^^,^^15^ Therefore, the two hypotheses are not mutually exclusive, and their proposed explanatory mechanisms seem to work at two different levels—proximate (e.g., byproduct of increased social tolerance) and ultimate (e.g., facilitating allomaternal care).

Both self-domestication and cooperative breeding hypotheses highlight the importance of social tolerance but consider it at the group level; consequently, these hypotheses potentially restrict prosociality to be a feature of socially tolerant species. Regarding the fitness benefits of prosocial preferences, the cooperative breeding hypothesis relies heavily on kin selection as in close kin groups of most cooperatively breeding species, prosocial preferences can produce indirect fitness benefits for the actor. Alternatively, however, there might also be direct fitness benefits of prosocial preferences among cooperative breeders, which also account for unrelated helpers; like increased survival when remaining in the group and subsequent pay-to-stay mechanisms, as well as increased access to current or future mating opportunities.^16^ Similarly, among self-domesticated and often colonial species, prosocial preferences may facilitate living in close proximity and may advertise quality, potentially leading to enhanced opportunities for (extra-pair) copulations (cf.^10^). However, even among these cooperatively breeding and self-domesticated species, prosocial preferences may vary across individuals (i.e., not all individuals show prosocial preferences)^17^ or contexts (e.g., audience presence:^18^), in line with some of the ultimate hypotheses. Nevertheless, variations in inter-individual prosocial motivations, even among cooperatively breeding (e.g., marmosets, azure-winged magpies, humans) or self-domesticated (e.g., dogs, humans, bonobos) species, are poorly understood.

The potential extent of interdependence is ample and can be maintained by mechanisms like kin selection and direct reciprocity. Despotic societies are generally characterized by low group-level social tolerance and high dominance asymmetries.^19^^,^^20^ Yet, despotism, particularly in Cercopithecines, favors kin, i.e., genetically closely related yet philopatric individuals (typically females) may gain adequate benefit out of nepotistic biases.^19^ Also, social support and coalition formation are evident among philopatric individuals in these matrilineal societies which may help increase rank in the hierarchical system,^21^ which might also be dependent on dyadic social tolerance levels and maintained through reciprocal exchanges.^19^^,^^22^^,^^23^ Therefore, selective prosocial preferences or biases could be expected among these reciprocating partners from a “Machiavellian Intelligence” perspective,^22^^,^^23^ in addition to such preferences among kin members. Besides, there is some empirical evidence present for prosocial preferences in relatively despotic societies, both in and outside of Cercopithecines, such as long-tailed macaques,^24^ capuchin monkeys,^25^ and chimpanzees^26^^,^^27^^,^^28^ (but see^29^^,^^30^^,^^31^). In the study with captive long-tailed macaques,^24^ individuals had the option of either granting food to a conspecific or obtaining the same for themselves. Individuals provisioned food more to kin than non-kin members, yet interestingly, the provisions were also dependent on the actors’ ranks in relation to non-kin partners. However, another study on captive long-tailed macaques concluded that prosocial preferences are not sufficient to exhibit costly prosociality (i.e., when the actor had to give up their own rewards) even among kin relatives.^32^ Using a dyad-specific barpull paradigm, unrelated capuchin monkeys showed prosocial preferences even in situations of slightly disadvantageous inequity.^25^ In contrast to these dyad-specific experimental studies, a study on chimpanzees found varying prosocial preferences using a “fountain task” in a group setting, where an individual could press a button leading to provisioning fruit juice to group members.^26^ The study also reported a positive link between social tolerance and the varying prosocial motivations in three different groups. These results, therefore, were obtained using different experimental set-ups and procedures and also differed from studies that found evidence of prosocial preferences in cooperative breeding and self-domestication species.^8^^,^^10^^,^^14^^,^^15^

Hence, testing more despotic species using similar set-ups and procedures is crucial for making clear inferences. Nevertheless, although the findings of prosocial preferences among individuals in relatively despotic species indicated a nepotistic influence, such tendencies toward non-kin members were also observed.^24^^,^^26^^,^^27^ Furthermore, these authors stress examining multiple groups due to evident inter-group variability in prosocial motivations among individuals; and investigating species that show even steeper dominance hierarchies, as there is a great lack of studies in despotic species where selective prosocial preferences can potentially be favored.

**Figure 1:**
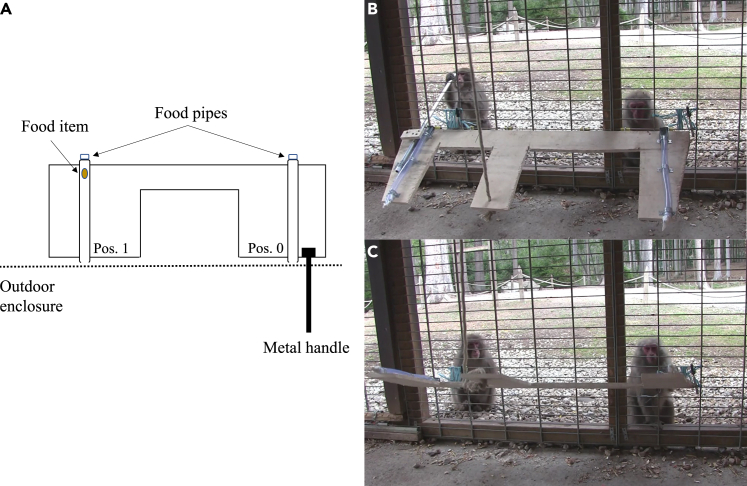
Experimental set-up of the group service paradigm (A) In this seesaw mechanism, a wooden board (length ∼1.5 m) had two transparent plastic pipes (Ø ∼ 3 inches) attached to two extremes (Pos. 0 or providing end and Pos. 1 or receiving end) through which food rewards (i.e., peanuts) could move. By default, the board was tilted toward the experimenter present inside the research hut (step (B) in the figure). A metal handle was connected to the board (next to Pos. 0) and projected outside, which upon pressing, could tilt the board toward the macaques (step (C)), resulting in food rolling down through the pipe(s) (if present) and coming in reach for individuals. Only an attempt to press, i.e., the release of the handle halfway, caused the seesaw to move back to its original position, and eventually, food (if present) would roll out of reach of the individuals again. Therefore, it was essential to fully press and hold the handle to get the food rewards. Notably, an individual pressing and holding the handle could not reach Pos. 1 due to the length of the board. (B and C) The consecutive steps show how an adult male Japanese macaque is providing food to an unrelated adult female. In the *test* condition, an individual needed to press the handle in Pos. 0 to make food accessible for another individual in Pos. 1. In the *empty control* condition, the experimenter pretended to place a food reward in Pos. 1. The access to the food pipe in Pos. 1 was blocked by attaching plexiglass to the enclosure in the *blocked control* condition; Food reward was thus visible but not accessible to the individuals, even if actors pressed the handle. For details, see STAR Methods.

Japanese macaques (*Macaca fuscata*) are a highly despotic species with remarkably low group-level social tolerance and high dominance asymmetry.^19^^,^^33^ Yet, they exhibit strong matrilineal bonds with clear signs of nepotistic biases.^34^ Moreover, coalition formation^35^ or alternate social strategies have been found to increase individual benefits within the hierarchy (e.g., male coalitions^36^). Besides, females, originating from large matrilines can compete with their sisters and subsequently form non-kin alliances with other individuals to “socially” outrank their higher-ranked sisters^37^; however, note that these results were found in artificially constructed group compositions, and it is unclear to what extent they can be generalized. In addition, Japanese macaques are capable of cooperative problem-solving in experimental settings in both semi-free and wild environments,^38^^,^^39^ for which considerable social tolerance is considered to be a pre-requisite.^40^ Selective prosocial preferences can therefore be beneficial, yet, previous studies found no evidence of such preferences in Japanese macaques in captivity^8^^,^^15^; using a group service food provision paradigm,^8^^,^^10^^,^^14^^,^^15^^,^^41^ prosocial provisioning was found to be 0%. The current study uses the identical group service paradigm (see Figure 1 for a brief overview) to investigate whether Japanese macaques in a semi-free-ranging condition have prosocial preferences. Our research offers several advantages over the previous studies on (Japanese macaque) prosociality – (a) a semi-free-ranging group with naturalistic conditions, i.e., high ecological relevance, (b) a relatively large sample size; i.e., a group of over 170 macaques, out of which 25 participated in our experiment voluntarily (see Table S1), (c) individual-level representation of prosocial preferences (if any), and (d) group level-as well as dyadic social tolerance measures. We hypothesize that the complexity of a large despotic society creates interdependencies among its members, favoring selective prosocial preferences among close kin members (i.e., matrilines) and among non-kin members with high social tolerance levels (i.e., potentially through direct reciprocity). We, therefore, expect that the likelihood and magnitude of food provisioning would be positively predicted by kinship and dyadic social tolerance.

## Results

### Group and dyadic social tolerance

The group-level social tolerance was measured by looking at the evenness of food distribution. Phase 2 of the group service paradigm was explicitly designed to do so (see STAR Methods and Video S1 for details). We calculated Pielou’s *J′*^14^^,^^15^^,^^42^ and obtained a value of 0.13, suggesting a highly uneven distribution of food (see Figure S1), and thus, a low group-level social tolerance.^8^^,^^10^ On the other hand, we found a dyadic social tolerance (see Video S2) range from 0 to 18 using the string-pulling task.^40^^,^^43^

Video S1. Group-level social tolerance test during Phase 2 of the group service paradigm, related to STAR Methods Individuals were obtaining food rewards from Pos. 1 of the apparatus. The alpha male approached and displaced others and started monopolizing.

Video S2. Dyadic social tolerance measure from the string-pulling task, related to STAR Methods Two individuals were sitting next to each other and obtained food rewards from the two ends of the wooden board. As the task required no joint action, one individual could pull the string and move the board.

### Group- and individual-level presses

We conducted five *test*s and five *empty control* (see Video S4) sessions, alternately, each containing 125 trials (which reflects 5 trials per participating animal, n = 25, cf.^4^^,^^6^). Similarly, we performed five *blocked control* sessions (see Video S5), alternating them with *empty (blocked) control* sessions, each consisting of 125 trials. Thus, individuals were presented with the opportunity to learn the contingencies of the procedure over five consecutive sessions per condition (see STAR Methods for details).

**Figure 2:**
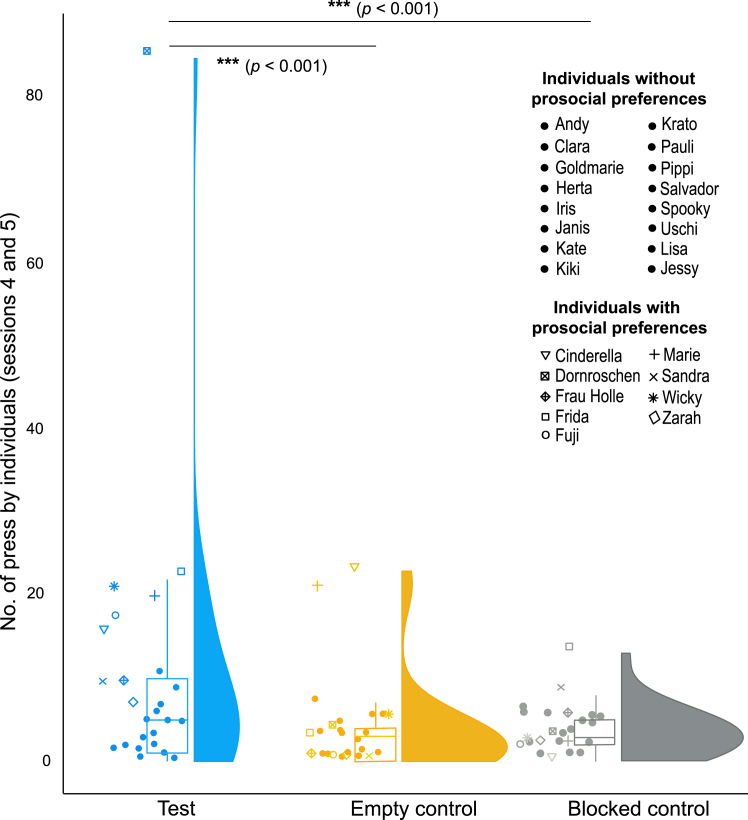
Overview of the number of presses across different experimental conditions Half-violin plots indicate the distribution of the data. Solid dots indicate the raw values of the individuals without prosocial preferences, whereas different symbols denote the raw values of the individuals with prosocial preferences (see identities in the figure). The boxes illustrate the interquartile range, horizontal bars inside the boxes indicate median values and whiskers indicate the range of the data, excluding outliers. ∗∗∗p < 0.001 (generalized linear mixed model (GLMM) with a Poisson log link distribution).

Based on responses from the final two sessions,^8^^,^^10^^,^^14^^,^^15^ we found a difference in terms of pressing the handle across different experimental conditions (percentages of presses in test: 88%, empty control: 36%, blocked control: 35%). The full model containing condition was found to be a better fit than the null model which had no fixed effects (likelihood ratio test/LRT: χ^2^ = 115.96, p < 0.001). Individuals pressed the handle significantly more often in the test, where food was in turn also delivered to a conspecific than in the empty- (GLMM: z = −8.365, p < 0.001, see Table S3) and blocked control- (GLMM: z = −8.589, p < 0.001, see Table S3) conditions (see Table S3, Figure 2), where there was either no food or where the access to the food for the recipient was blocked, respectively.

**Figure 3:**
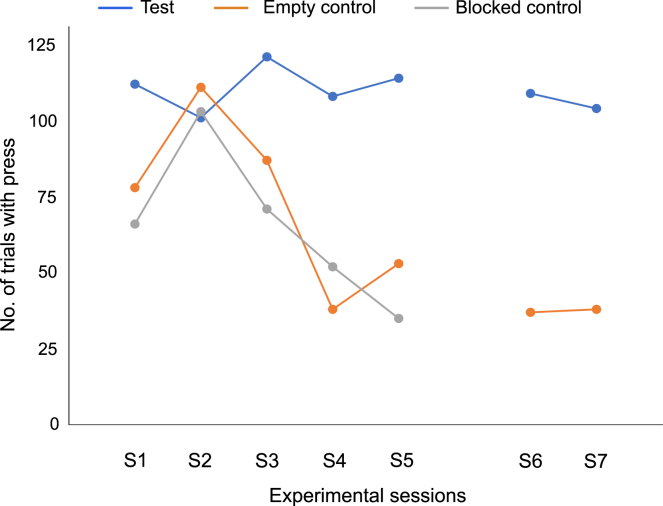
Trials with presses across sessions and experimental conditions Out of the predefined 125 trials, trials with presses differed across experimental conditions and sessions (GLMM with a negative binomial distribution). S1-S5 are regular sessions, while S6 and S7 indicate re-test and re-empty control sessions. The number of presses was comparable between regular test and re-test (85.2%), and regular empty control and re-empty control (30%) sessions (GLMM with a negative binomial distribution).

As the blocked control sessions were conducted after both the test and empty-control sessions (alternating with each other), we conducted two re-test and re-empty control sessions (again alternately) to account for order effects. During re-test and re-empty control sessions, the percentage of presses (test = 85.2%, empty control = 30%) was comparable to earlier sessions (test = GLMM: z = 1.167, p = 0.24, see Table S4; empty control: GLMM: z = 0.511, p = 0.61; see Table S5, Figure 3), indicating no bias due to the pre-determined order of the experimental phases (cf.^14^).

Finally, a sustained yet high rate of press across all *test* sessions (sessions 1–5) was observed, i.e., the rate of the press did not differ across sessions (see Table S6). This was observed in contrast to the pattern of declining rates of press in empty- and blocked-control sessions (Figure 3). These results thus indicate that the animals first had to learn the contingencies of the controls, whereas their prosocial preferences seemed to be present from the beginning and remained stable over the 5 regular- and 2 re-test sessions.

At the individual level, we found three macaques who pressed the handle significantly more often in the test than in both control conditions. Note, however, that the potential amount of test trials (where prosocial preferences can be observed) in which each individual could press (125 trials/session) was restricted by the number of participating individuals and was also dependent on the number of presses by other individuals. Consequently, pressing significantly more in the test than in both control conditions was a particularly conservative cut-off for showing differentiation of the conditions at the individual level. Therefore, we also considered individuals (n = 7) who pressed significantly more often in the test than in at least one of the control conditions ^cf.^^10^^,^^14^ (empty control—five individuals; blocked control—two individuals), to be able to differentiate between the different conditions and to understand that pressing in the test situation would result in a reward for someone else. Notably, all 25 participating individuals passed the initial phases of the experiment and experienced the first three sessions of each condition and should thus have some general knowledge about the contingencies. A lack of significant differences for the remaining individuals may therefore also reflect a lack of motivation to provide to others rather than a lack of understanding. Alternatively, individuals who never pressed in either control condition, and only a few times in the test condition do show a clear understanding of the task but maybe do not have prosocial preferences, which was the case for one of our individuals (number of presses per condition: test = 10, empty control 0, blocked control = 3). This then left us with 9 individuals who we considered to have prosocial preferences compared to others that we considered to have none (see Table S2, Figure 2).

### Latency to press the handle

In an initial linear effect model (LM), we found a significant interaction effect between the two fixed effects, i.e., sessions and experimental conditions (see Table S7). However, we found a variable inflation factor (VIF) value of 5.92 for the interaction, suggesting collinearity. Therefore, in the next model, we dropped the interaction term and investigated the main effects. The new model differed significantly from the null model (LRT: χ^2^ = 118.43, p < 0.001). The latencies of pressing the handle were found to be different across the three experimental conditions (see Figure S2). Individuals pressed the handle faster in the test condition (18.32 ± 22.31 s) as compared to both empty (45.37 ± 29.18 s, LM: t = 9.584, p < 0.001, see Table S7) and blocked (44.52 ± 32.18 s, LM: t = 9.047, p < 0.001, see Table S7) control conditions. We did not find any effect of sessions (LM: t = −1.032, p = 0.30, see Table S7). Note that here, we only considered trials when there was a press; therefore, trials without a press were discarded from this analysis.

### Food provision

Overall, food provisions, i.e., presses that led to a recipient getting the food, occurred in 72.8% of the trials by all individuals (n = 25). Notably, even though a specific individual pressed more often in the test than in empty control, she did not successfully provide any food to others. As mentioned above, we excluded her from the list of individuals with prosocial preferences due to a lack of clear evidence of food provisioning. A more conservative measure of total prosocial provisioning was subsequently applied, where we corrected the percentage of provisioning based on the number of individuals with prosocial preferences (i.e., nine individuals) who seemed to understand the consequences of their actions in the group-service paradigm, and the resulting percentage of trials in which food was provisioned to a conspecific was 68.8%.

Although the individuals with prosocial preferences seemed to provision food at a sustained rate (see Figure S3), there was a significant difference between session one (no. of provisions ±SD: 3.9 ± 9.02) and five (8.8 ± 9.67) of the test condition (GLMM: z = 2.305, p = 0.02, see Table S8), indicating an increase in food provision from session one to five. The sustained pressing by these individuals across sessions suggests their intent to benefit others remained stable, yet the increased subsequent food provision rates may reflect better coordination between the actor and receiver in later sessions.

Furthermore, we compared the number of food provisions and food received by the individuals with prosocial preferences (see Table S9). These individuals provided food to others (no. of provisions ±SD: 19.11 ± 22.83) significantly more than the amount they received (4.11 ± 3.58, Wilcoxon signed-rank test: z = - 2.43, p = 0.01). This may indicate that these individuals indeed cared for provisioning group members rather than obtaining rewards for themselves through (direct) reciprocity.

### Aggression and solicitation

We found very few instances of aggression from actors to receivers (6.59%) after provisioning food (goodness of fit chi-square test: χ^2^ = 137.16, p < 0.001). Also, active solicitation in the form of reach-out behavior (i.e., holding the food pipe in Pos. 1 by extending the arm(s)) was observed (16.48%) in significantly few instances by receivers toward actors (goodness of fit c5828hi-square test: χ^2^ = 81.78, p < 0.001). These results indicate that the provisioning was proactive by the actors.

Furthermore, in 22% of the trials, individuals were already present within one arm’s distance from Pos. 1, i.e. at the receiving end, when an individual with prosocial preference pressed the handle and provided food. In all other trials, i.e. in a significantly higher number of trials (goodness of fit chi-square test: χ^2^ = 57.16, p < 0.001), the act of pressing the handle seemed more proactive and not induced by the presence of a partner, which may also explain why not every press resulted in food provisioning (notably, food provision occurred in 68.8% of the trials by individuals with prosocial preferences, where there was a press). This is not to say that there were no links between the actor and the recipient who ultimately received the reward.

### Kinship and dyadic social tolerance

To assess what might explain the likelihood and magnitude of food provisioning among different dyads, we examined the effects of kinship and dyadic social tolerance. It is important to note here that the 25 participating individuals belonged to eight different matrilines. Considering a large number of participating individuals (n = 25) and among individual variations in prosocial preferences, we analyzed the likelihood and number of food provisioning across all potential dyads, including at least one of the individuals who seemed to have some prosocial preferences. Assuming that an individual (n_prosocial_ = 9) could provide food to anyone except for themselves (i.e., potentially 24 individuals), a total of 9 x 24 = 216 potential dyads (i.e., individuals with prosocial preferences as actors and all participating individuals as receivers) were considered in further analyses.

**Figure 4:**
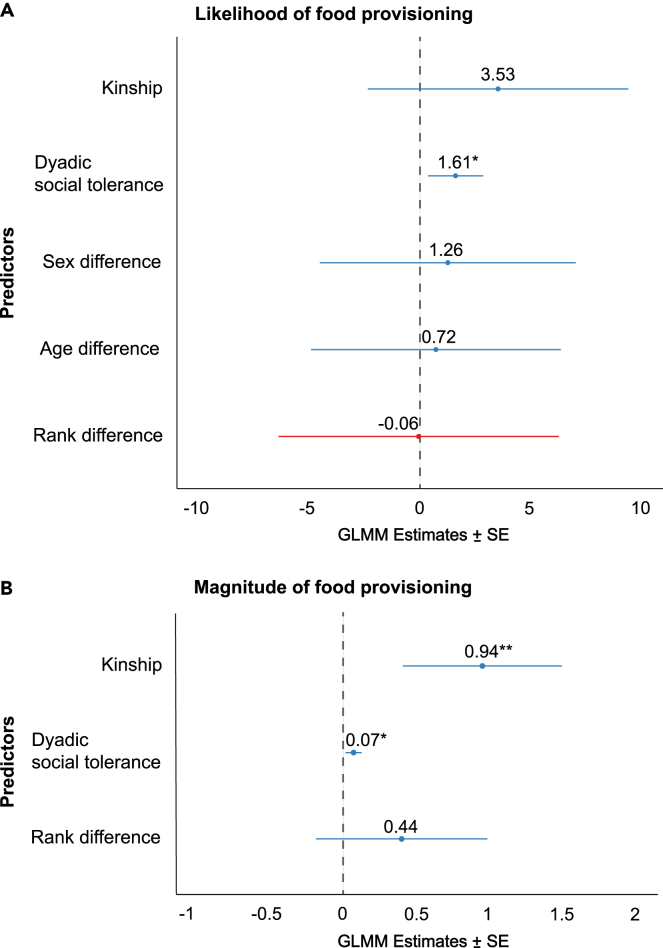
Model overview investigating the likelihood and magnitude of food provisioning GLMM coefficients ± SE of the models are presented. Blue lines indicate positive associations between the predictors and response variable, whereas the red lines indicate negative associations; the vertical dashed line indicates no effect. (A) We found only a positive effect of dyadic social tolerance on the likelihood of food provisioning. (B) Both kinship and dyadic social tolerance positively predicted the magnitude of food provisioning. Note, food provisioning was restricted to (i.e., as actors) individuals with prosocial preferences. ∗∗p < 0.01, ∗p < 0.05 (GLMM with a binomial distribution for likelihood- and GLMM with a Poisson distribution for magnitude of food provisioning).

We found that the likelihood of food provisioning was positively predicted by dyadic social tolerance (GLMM: z = 1.610, p = 0.01; see Table S10, Figure 4A); no effect of kinship (GLMM: z = 3.534, p = 0.24, see Table S10, Figure 4A) was found. We did not find any effect of age- (GLMM: z = 0.251, p = 0.80, see Table S10), sex (GLMM: z = 0.426, p = 0.67, see Table S10) and rank (GLMM: z = −0.02, p = 0.98, see Table S10) differences. This full model significantly differed from the null model (LRT: χ^2^ = 79.96, p < 0.001). None of the fixed effects in the full model showed collinearity. In a substitute model, we inspected the age and sex differences in detail, where all possible directions were included (age differences: provisions from - adult to juvenile, juvenile to adult, adult to adult, and juvenile to juvenile; sex differences: female to male, male to female, female to female, and male to male). However, the results did not change (see Table S11), therefore, we kept the first full model as the best-fitted model.

Both kinship (GLMM: z = 2.984, p = 0.002; kin = 3.68 ± 7.18; non-kin = 0.32 ± 1.41; Figure 4B) and dyadic social tolerance (GLMM: z = 2.062, p = 0.03; Figure 4B) were found to influence the magnitude of food provisioning, independently. The best-fitted model included dominance rank difference, in addition to kinship and dyadic social tolerance, as fixed effects (see Table S12). We found this model to be significantly different from the null model (LRT: χ^2^ = 79.96, p < 0.001). However, no effect of dominance rank difference was found (GLMM: z = 1.307, p = 0.19). We did not find any collinearity among the fixed effects. Similar to the likelihood approach, a substitute model was used with detailed age and sex differences; however, the results remained the same (see Table S13). Therefore, we retained our first model as the best-fitted model.

## Discussion

We examined whether individuals in a highly despotic society, like Japanese macaques, have prosocial preferences. In contrast to previous findings on small captive populations (where the percentage of prosocial individuals was null), our study, using a much larger semi free-ranging population of Japanese macaques, provides evidence of prosocial preferences among 36% of the participating individuals. In a species that is neither self-domesticated nor cooperatively breeding, individuals with such preferences provisioned food at considerably high levels, i.e., in 68.8% of the test trials. However, only 9 out of the 25 participating individuals (i.e., 36%) did so, suggesting substantial individual-level variation. At the same time, it is important to note that despite the differential objectives of the two control conditions (empty control: stimulus enhancement; blocked control: food motivation), we considered individuals to have prosocial preferences who pressed the handle (and provisioned food) more often in the test than in at least one of the control conditions. This is due to the presence of a potential “ceiling effect,” i.e., the constraint of the number of trials available for the participating individuals in each condition. Nevertheless, the evidence of food provision was highlighted as we put more emphasis on the observed instances of food provision over pressing the handle. Although we found a clear kin bias in the magnitude of food provisioning, such provisions were not restricted to kin-relatives, as non-kin dyads with relatively high social tolerance were also found to provide food to each other. Also, the low aggression and solicitation rates in our results indicate the proactive and intentional nature of food provisioning by individuals with prosocial preferences.

### Genuine prosocial intent

Burkart and van Schaik^15^ illustrated that in order to call food provisions proactive, some criteria need to be fulfilled in the group service paradigm—higher frequency of presses in the test than in controls, sustained or emerging provisions throughout the test condition, and provision in the absence of solicitation. The prosocial intent of 9 out of the 25 participating individuals in our study group of Japanese macaques seemed genuine as they fulfilled all the above criteria.

Evidently, the above-mentioned individuals pressed the handle more often in the test than in the control conditions. Unlike the decreasing pattern of pressing rates in both control conditions over the subsequent sessions, the sustained or increased rates of pressing in the test condition testify that individuals understood the contingencies of the experiment well. A faster response time to press the handle in the test than control conditions further supports the statement. Individuals were equally motivated throughout the study, independent of test or control condition, as seen from their responses in the motivation trials (96% success). Therefore, the low press rates during control conditions were not governed by the lack of willingness to participate. Moreover, the negligible amount of aggression from actors to receivers could explain the prosocial preferences of the macaques, rather than trying to procure the food rewards themselves, albeit impossible. In addition, the individuals with prosocial preferences provided food significantly more to others than what they received, suggesting their non-reliance on immediate reciprocal benefits.

The fact that active solicitation and the presence of individuals being potential receivers did not necessarily result in food provisioning indicates its proactive nature. For instance, active solicitation, begging or signal prompting has been argued to be a stimulus enhancement mechanism of rather “reactive” prosociality.^27^^,^^40^^,^^41^ In contrast, some studies even found a negative role of signal prompting on helping behavior.^44^^,^^45^^,^^46^

### Kin favoritism and the influence of dyadic social tolerance

Kin favoritism and nepotism are integral properties of Japanese macaques and, in general, despotic cercopithecine societies.^19^^,^^47^^,^^48^ Our study group followed species-typical matrilineal norms, i.e., the presence of distinct hierarchical ranks and order.^34^^,^^49^^,^^50^ As hypothesized, kinship was found to influence food provisioning, though it did not predict the propensity or likelihood of the same. This might suggest a potential role of direct reciprocity at play with regard to the positive effects of dyadic social tolerance among group members. Nevertheless, we did find evidence of kin relationships positively influencing the magnitude of provisioning. That is, the majority of the food provisioning was restricted to kin members. These findings align with the kin-selection theory,^51^ i.e., prosocial preferences among kin members can lead to higher indirect benefits.^52^ Besides, even though the chances of future reciprocity^53^ can be expected, the actors at the time of helping might not be certain about such returns, explaining provisioning kin members with unselfish motives from a proximate point of view.^54^

Close kin membership can be instrumental in social support and interdependencies, especially in despotic societies with frequent aggression and conflicts.^50^^,^^55^^,^^56^^,^^57^ Therefore, prosocial preferences among kin members can be highly beneficial. In line with this, prosocial preferences were also found among close kin members in the slightly less despotic long-tailed macaques.^24^ However, it is crucial to disentangle the roles of kinship and dyadic social tolerance, i.e., whether they essentially foster each other or are mutually exclusive. Our study neither found an interaction effect of kinship and dyadic social tolerance facilitating prosocial preferences nor collinearity between the two. Therefore, it could be possible that the linear hierarchical system is at play, leading to lower social tolerance levels even among the members of the same matriline than conventionally thought. We speculate that the dyadic social tolerance task could not fully capture the different facets of relationship quality,^58^ or have represented a completely distinct mechanism, e.g., direct reciprocity, another key factor influencing prosocial preferences.

Nevertheless, we found a positive effect of dyadic social tolerance on food provisioning that may be explained through direct reciprocity and can favor social support and alliance formation among unrelated group members. However, note that calculated future reciprocity is cognitively demanding^59^ and unlikely to be maintained in non-human primates.^60^^,^^61^ Thus, complexity in a despotic society like Japanese macaques can promote interdependencies where reciprocal exchanges might not require strict bookkeeping yet still be maintained by behaviors like grooming and other affiliative interactions. Despite the strong predictive value in reciprocal exchanges in a range of species,^39^^,^^42^^,^^58^^,^^59^^,^^60^^,^^61^ there is a great lack of studies concerning dyadic social tolerance.^40^^,^^43^^,^^62^^,^^63^^,^^64^^,^^65^

### The evolution of prosociality, the influence of socio-ecological conditions, and the “interdependency hypothesis”

So far, there have been two influential hypotheses explaining the phylogenetic differences in prosocial preferences across taxa—the self-domestication hypothesis^11^ and the cooperative breeding hypothesis.^8^^,^^13^^,^^15^ Japanese macaques are neither (self-) domesticated nor cooperatively breeding. Yet, interestingly, the observed food provisioning rate (∼69%) of the Japanese macaques in our study resembled cooperative breeding and self-domesticated species’ provisioning,^8^^,^^10^^,^^15^ notably while using the exact same set-up and procedure. These results, together with the findings of prosocial preferences (although using different set-up and/or procedures) among other non-domesticated species and species that lack cooperative breeding or allomaternal care,^24^^,^^26^^,^^27^^,^^66^^,^^67^^,^^68^ call for additional hypotheses explaining the evolution of prosocial preferences.

Both the cooperative breeding and the self-domestication hypotheses, to a reasonable extent, highlight dependencies among individuals and predict high social tolerance within a group. Our study group, as expected given their despotic social structure,^69^ showed very low group-level social tolerance, but we did identify varying dyadic tolerance values, i.e., potential interdependencies (e.g., alliances) among its members. Thus, despotic societies, like cooperatively breeding and self-domesticated species, may too provide opportunities for individuals to have prosocial preferences but selectively, which can be maintained through kinship and direct reciprocity. We, therefore, call for a more general “interdependency hypothesis” to explain the evolutionary origins of prosocial tendencies and the phylogenetic differences observed. Note that interdependency has also been postulated as an explanatory variable for variation in inequity responses across and within species.^70^

Our results question the premises of the covariation framework or correlated variations across macaque species in social traits (such as asymmetry of resource holding, kin-favoritism and biases, counter aggression, and reconciliation, etc.),^19^^,^^33^ as it expects little to no cooperative behaviors in despotic species with low group-level tolerance. We argue here that instead, in such despotic societies, cooperation may be paramount to attain and maintain rank positions and that it, as such, creates interdependencies favoring selective prosocial tendencies (cf. “Machiavellian Intelligence”,^22^^,^^23^).

### Between-group variation in Japanese macaques’ prosocial tendencies

Our results are in stark contrast with previous reports on prosocial preferences in captive Japanese macaques (∼0%,^15^). Consistent with the prediction that independently breeding free or semi free-ranging primates have lower social tolerance than captive populations,^71^^,^^72^^,^^73^^,^^74^ our study group showed lower group-level social tolerance (Pielou’s *J′* = 0.13) than the previously tested population residing in a more restricted captive setting (Pielou’s *J′* = 0.32).^15^ It must be noted that our group-level tolerance measure was limited to focal individuals and not the entire population. Nevertheless, this again suggests that group-level social tolerance may not be a good predictor of prosocial preferences, especially when such preferences are selective. Yet, striking variation at the dyadic social tolerance level was observed among kin and non-kin group members, and as discussed above, they did predict the likelihood and magnitude of food provision in our population. Interestingly, in the above-mentioned study on captive Japanese macaques,^15^ even though the conservative percentage of provision was reported as null, few food provisions were noticed in the initial sessions of the test condition. Out of the 16 successful provisions by individuals with “prosocial preferences,” 13 of them were restricted to a mother-daughter pair, which may also indicate the role of kinship and may exhibit high-dyad specificity. It is also important to note that out of the 10 individuals present in that group, only 6 individuals participated. These contrasting results between the two Japanese macaque populations could thus potentially be attributed to the more complex socio-ecological conditions (i.e., multiple matrilines, larger group size, varying age and sex class of the individuals, higher opportunities for reciprocity, and subsequent degrees of tolerance) of the semi free-ranging group, leading to selective prosocial preferences among individuals.

Relatively large size and complexity of a group can drive cognitive processes, as proposed by the social intelligence hypothesis,^23^^,^^75^^,^^76^ even within single species.^77^ Such complexity might create opportunities for individuals to become “Machiavellian” and make them take “political” decisions.^75^^,^^78^ Subsequently, prosocial preferences for specific partners through kinship and potential reciprocity can facilitate support, especially during conflicts and coalitions.^78^^,^^79^ A classic example in the current study would be the beta male providing food to the beta female (Video S3). Thus, maintaining alliances with other high-ranked individuals could help the beta male rank up in the hierarchy and become the alpha.

Video S3. Group service test condition, related to STAR Methods The adult male in Pos. 0 pressed the handle, and as a result, the seesaw tilted. The adult female sitting in Pos. 1 obtained the food reward.

Video S4. Group service empty control condition, related to STAR Methods The experimenter pretended to place food in Pos. 1. The individual did not press the handle within the duration of the trial, suggesting an understanding of the contingency of the control condition.

Video S5. Blocked control condition, related to STAR Methods The access to Pos. 1 was blocked using transparent plexiglass. Individuals noticed the food reward placed but could not obtain it. The individual did not press the handle within the duration of the trial, suggesting an understanding of the contingency of the control condition.

Finally, prosociality has been argued to be the foundation for inter-group conflict^80^; consequently, in-group prosociality and parochial cooperation can be favored. Our study group recently underwent fission, forming two distinct subgroups,^81^ and all participating individuals in the current study belonged to one of the subgroups. Therefore, it would be interesting to test in future studies whether such an ecological event of fission may have governed the prosocial motivations we witnessed in our study.

### Conclusion

We provide the first experimental report of selective prosocial preferences in semi free-ranging Japanese macaques. We discussed how kin favoritism and dyadic social tolerance (potentially through reciprocity) in a despotic society could maintain individual prosocial motivations.^33^ While we did not find the age difference of the individuals to influence food provisioning significantly, the participation of a relatively large number of juveniles cannot be neglected. For instance, due to their elevated social tolerance, bonobo sub-adults have been shown to exhibit higher prosocial preferences than adults.^41^ Nevertheless, our findings are not entirely restricted to the predictions of the cooperative breeding hypothesis^9^ (also see^82^^,^^83^) and the self-domestication hypothesis,^7^ thus, we emphasize inter-individual dependencies other than allomaternal care to be valued and included in an overarching “interdependency hypothesis,” which can systematically be tested using an evolutionary framework. Finally, as previously stated by other researchers, we would also like to stress exploring multiple groups of a species with similar and different living conditions (i.e., captive, semi free-ranging, and free-ranging) and structures, to come to a conclusion on that species’ prosocial tendencies, including selectivity in responses. Similarly, it might be interesting to see whether population differences in prosocial preferences can be a result of potentially differential social cultures in these populations.^84^

### Limitations of the study

Although our study generates contrasting and unprecedented results on prosocial preferences from a large group of despotic Japanese macaques, we do not entirely discard the possibility of potential group-level differences. Therefore, the study would have benefitted from comparing the current findings with another semi free-ranging group of Japanese macaques. Additionally, only a small percentage of the population (25 out of 170 individuals) voluntarily participated in the task; of which, 9 individuals exhibited prosocial tendencies. The participants thus might be considered “bold” and/or “explorative” compared to other group members, leading to a potential sampling bias. Nevertheless, here we prioritized ecological relevance and welfare, as no forced dyads were formed. Finally, we used an experimental approach to measure dyadic social tolerance, which, even though a powerful proxy of social relationships,^40^^,^^43^ could not be validated with long-term observational data, especially using natural co-feeding behaviors.

## STAR★Methods

### Key resources table

**Table:** 

REAGENT or RESOURCE	SOURCE	IDENTIFIER
**Deposited data**		

Raw data and code	This paper	Dryad Dataset: https://doi.org/10.5061/dryad.kh18932b2

**Experimental models: Organisms/strains**		

Japanese macaques (*Macaca fuscata*)	Affenberg Zoobetriebsgesellschaft mbH, Austria	N/A

**Software and algorithms**		

R (version 4.2.2)	R Core Team^85^	https://www.Rproject.org/
Adobe Illustrator (version 25.3)	ADOBE	https://www.adobe.com/products/illustrator.html

**Other**		

Video Camera (Canon Legria HF G25)	CANON	https://www.canon.nl/for_home/product_finder/camcorders/high_definition_hd/legria_hf_g25/

### Resource availability

#### Lead contact

Further information and requests for resources should be directed to and will be fulfilled by, the lead contact, Debottam Bhattacharjee (bhattacharjee.debottam@gmail.com).

#### Materials availability

Besides data and R codes, this study did not generate any new reagents or materials.

### Experimental model and subject details

The study was conducted with a semi-free-ranging group (population size ∼170 individuals) of Japanese macaques (*M. fuscata*) at Affenberg Landskron (latitude 46°38′32.51″N; longitude 13°53′48.98″E) in Austria from February to May 2022. The macaques were present in an enclosure of 40,000 m^2^ with a natural mixed forest common to southern Austria^49^ (see Figure S4). At the time of testing, approximately 74% and 19% of the individuals were >3 and ≤3 but ≥1 year old, respectively (see^81^ for details). The study group was habituated to the presence of human experimenters but with limited human intervention, except for feeding and emergency medical purposes (such as veterinary care).

Out of the 170 individuals, a total of 25 macaques (nine individuals ≤3 but ≥1 year old; sixteen individuals >3 years of age) voluntarily participated in the study and self-trained themselves (see below) while remaining in their social group (see Table S1). Out of the 25 individuals, 19 were female and 6 were male (see Table S1). Thus, no individuals were forced to participate in the experiments. We obtained the matriline ranks and information on dominance relationships from a previous study on this population^49^ (see Table S1). All individuals from the same matriline were related to a certain degree and thus were considered kin relatives (see Table S1).

The study complied with Austrian Law (§ 2. Federal Law Gazette number 501/1989) and Code for Best Practices in Field Primatology. The approval for the study was obtained from an internal board of the Austrian Research Center for Primatology (ARCP), which also oversaw the whole process. All the components of the study were non-invasive, requiring no ethical approval according to the European Directive 2010/63. We strictly followed the ethical principles and guidelines of the American Society of Primatologists for the care and inclusion of animals in our study.

### Method details

#### Experimental design: Group service paradigm

We adopted a modified version^10^ of the group-service paradigm.^15^ The procedure included several phases occurring in the following order – Phase 0 (habituation to apparatus), Phase 1 (initial training and habituation to procedure), Phase 2 (food distribution assessment), Phase 3 (apparatus training), Phase 4 (group-service), Phase 5 (blocked control), and Phase 6 (Re-test). Phases 0, 1 & 3 consisted of habituation and training sessions. It is important to note that 25 individuals (adult female = 13, adult male = 3, juvenile female = 6, juvenile male = 3, see Table S2) successfully passed the criteria of phases 1 and 3 and that the number of trials in all other phases was based on this number of participants, i.e. n ∗ 5 = 25 ∗ 5 = 125 trials.

A seesaw mechanism was used (Figure 1), where a wooden board (length ∼1.5 m) had two transparent plastic pipes (Ø ∼ 3 inches) attached to two extremes (Pos. 0 or providing end and Pos. 1 or receiving end) through which food rewards (i.e., peanuts) could move. By default, the board was tilted toward the experimenter present inside the research hut (Figure 1B). A metal handle was connected to the board (next to Pos. 0) and projected outside, which upon pressing, could tilt the board toward the macaques (Figure 1C), resulting in food rolling down through the pipe(s) (if present) and coming in reach for individuals. An attempt of press, i.e., releasing the handle halfway, caused the seesaw to move back to its original position, eventually making food (if present) out of reach of the individuals again. Therefore, it was essential to fully press and hold the handle to provide the food rewards. Notably, an individual pressing and holding the handle could not reach Pos. 1 due to the length of the board (∼1.5 m).

During the habituation phase (Phase 0), food items (i.e., shelled peanuts) were spread over the wooden board at regular intervals. This phase was carried out to reduce and/or eliminate any potential neophobia toward the seesaw apparatus and to habituate individuals to the setup. The experimenter tried to catch the attention of the individuals by calling “Monkeys”. Three sessions were conducted (each on one day for ∼1 h). A total of 25 individuals participated in this phase voluntarily and obtained food rewards successfully, as mentioned above. In the initial training and habituation to the procedure phase (Phase 1), the mechanism of the seesaw was completely locked, and the platform was tilted toward the individuals. So, when placed in either position, food rewards rolled down to the individuals. We conducted a total of five sessions, and for each session, food was provided in Pos. 0 and Pos. 1, alternately. The number of trials was determined according to the number of individuals who successfully passed Phase 0 (i.e., 125 trials/session). This number was used for the following phases as well. A trial began when a food reward was placed (either in Pos. 0 or Pos. 1) and ended with an individual retrieving it or after 2 min. In this phase, an individual was considered trained if they received at least a total of 10 rewards throughout the different sessions. To fulfill the criterion, at least half of the habituated individuals needed to get trained. We recorded which individual(s) obtained the rewards for each trial. We tested the group-level social tolerance in the next phase (Phase 2). The procedure was the same as in phase 1, with the mechanism still locked and the platform tilted toward the individuals (see Video S1). However, food rewards were always placed in Pos. 1 in this phase. We conducted two sessions, with each having 125 trials. Since the apparatus training phase (Phase 3), the seesaw mechanism was operational. Therefore, individuals needed to learn to press the handle to tilt the board and get the rewards. Food rewards were always placed in Pos. 0. Thus, individuals pressing and holding the handle could make food rewards available to themselves. The phase was completed when at least half of the individuals obtained at least ten rewards over five sessions.

In the *test* sessions (Phase 4), we provided food rewards (shelled peanut) in Pos. 1 of the seesaw apparatus (see Video S3). We counted the number of instances individuals pressed the handle and provided food to group members. In addition to the test sessions, we conducted *empty control* sessions (Phase 4), identical to the test, except that no food was placed, even though the same movement (of placing food in Pos. 1) was made to control for stimulus enhancement (see Video S4). In the *blocked control* sessions (Phase 5), Pos. 1 was blocked using plexiglass, thereby making the food visible yet inaccessible to the individuals. The blocked control sessions investigated whether pressing in the test was only due to the presence of food (see Video S5); the rest of the steps were identical to the test sessions. Besides, two additional *tests* and two *empty control* sessions (Phase 6) were conducted alternately after the blocked control sessions, to eliminate any potential bias due to the order of the phases in the experiment.

A test trial began when a food reward was placed in Pos. 1 and ended with an individual retrieving it or after 2 min (see Video S3). In contrast, the control trials lasted for 2 min. In empty (see Video S4) and empty blocked control conditions, a trial began when the experimenter pretended to place a food reward in Pos. 1. For the blocked control condition (see Video S5), food was placed in Pos. 1 but was not accessible to the individuals. Similar to test, a trial began with the experimenter placing a food reward in Pos. 1 in the blocked control condition. All control conditions had motivational trials. The inter-trial time interval was within a range of 8–10 s. The trials were conducted only when at least two trained individuals were present within a radius of 5 m of the experimental setup. We found only two instances (out of all trials from Session 1 to Session 5 of the *test* condition), where an individual outside of the 25 ‘trained’ subjects received a reward. We conducted those two trials again.

For phases 4–6, in each session, be it *test* or *control*, we added motivation trials where food was placed in Pos. 0 at regular intervals, i.e. after every fifth trial. These trials were included to ensure low pressing rates in either test or control sessions (if observed) were not due to a lack of motivation to participate. Overall, the monkeys were very motivated as they pressed in 96% of these trials, independent of the experimental conditions (Test – Empty control, Goodness of fit Chi-square test: χ^2^ = 0, p = 1; Test – Blocked control, χ^2^ = 0.709, p = 0.67; Empty control – Blocked control, χ^2^ = 0.709, p = 0.67). Thus, motivation alone did not influence pressing the handle in either condition.

#### String-pulling task

For the string-pulling (dyadic tolerance) task, we used a movable wooden platform (length ∼1.5 m) to which two ropes were attached at the two extremes (see Video S2). Two pieces of highly preferred food rewards (peanuts) were placed out of reach of the macaques on two ends of the platform (∼1m apart). The platform could be moved by pulling either of the ropes. As this task did not require joint action, one individual could pull, move the platform and monopolize the two food rewards. Similar to the seesaw mechanism, we conducted a habituation phase with the string-pulling apparatus for an entire day (from 0930 to 1830 h). Individuals were considered habituated after obtaining at least five rewards from the wooden board. During the test trials, we recorded the individuals who ‘tolerated’ each other, i.e., simultaneously obtained the highly rewarding food items while sitting next to each other. A total of 18 sessions were conducted, each consisting of 20 trials.

### Quantification and statistical analysis

Identities of the individuals were noted live with regard to pressing the handle, providing and receiving food rewards, and confirmed later via videos by the experimenter. A second rater, who was not the experimenter, scored the behavioral variables for all the sessions. Inter-rater reliability was excellent (ICC(3,k) = 0.98).

The group-level social tolerance was measured by looking at the evenness of food distribution during Phase 2 of the group service paradigm (see Video S1). We calculated Pielou’s *J′*,^14^^,^^15^^,^^42^ an index which could range from 0 to 1, “0” being the lowest tolerance and “1” indicating a high tolerance with equal distribution of food resources among group members. Additionally, we counted the instances where two individuals retrieved food rewards simultaneously during the string-pulling task (see Video S2) to assess dyadic social tolerance between two specific individuals. The strength of dyadic social tolerance was defined by the number of instances where two individuals retrieved food rewards simultaneously without showing any signs of aggression.^40^^,^^43^

We ran a generalized linear mixed model (GLMM) with a Poisson distribution to investigate the number of presses across test, empty control, and blocked control conditions (based on sessions 4 and 5); the number of presses (cumulative press from sessions 4 and 5) was included as a response variable, experimental condition (test/empty control/blocked control) and identity of the individuals as fixed- and random effect, respectively. To check for a potential order effect, i.e., the pre-determined sequence of phases, in the experiment, we compared the responses of sessions 4 and 5 (test and empty control) with re-test (sessions 6 and 7) and re-empty control (sessions 6 and 7) sessions. Two separate GLMMs with negative binomial distribution were conducted for the number of presses in test-re-test and empty-re-empty control conditions. All other variables were identical to the previous model. Besides, a negative binomial GLMM was used to check whether the number of presses differed across the test sessions; sessions (sessions 1–5) were used as fixed effects and individual identities as a random effect in the model. In addition to the group-level investigation, we conducted Fisher’s exact tests for each individual and compared the presses of the test with empty- and blocked-control conditions. A Bonferroni correction method was applied for the post hoc analysis, and an adjusted p value of 0.025 was set as the level of significance. Moreover, we compared the latencies to press the handle across the three experimental conditions. In addition to conditions, we included sessions (4 and 5) in a LM. Similar to presses across sessions in the test condition, we also looked at whether the food provision differed. We used a negative binomial GLMM with sessions (1–5) as fixed- and individual identities as random-effect. Goodness of fit chi-square tests were conducted to compare trials with and without – (i) active solicitation from helper to actor, (ii) aggression from actor to helper, and (iii) presence of an individual at Pos. 1.

Finally, we used a hurdle model approach to investigate the likelihood and magnitude of food provisioning. For likelihood, we conducted a binomial GLMM (food provision: yes/no), whereas, in the latter, a Poisson GLMM was used (number of food provisions, i.e., >0). All possible combinations of dyads were built based on individuals with and without prosocial preferences and included as random effects (i.e., actor/receiver). Both models included kinship (yes/no) and dyadic social tolerance (count) as fixed effects. Also, sex, age, and rank differences (all categorical) were included as control variables.

All statistical analyses were carried out in R (4.2.2).^85^ The GLMM and LM analyses were conducted using *lme4*^86^ and *glmmTMB*^87^ packages. Null vs. full model comparisons were made for all the models using the ‘lrtest’ function of the package *lmtest*.^88^ We investigated the model residual distribution, dispersion and outliers using *DHARMa* package.^89^ The multicollinearity of the predictors was examined with the help of *performance*^90^ and *car*^91^ packages and a variance inflation factor (VIF) of <5 was set as a threshold for low correlation between the predictors.^92^ The significance value (α) was set as 0.05 for all statistical tests, except for the Bonferroni correction where it was set as 0.025.

## Data Availability

• All data generated in the study have been deposited at Dryad repository and are publicly accessible as of the date of publication. The DOI is provided in the key resources table. • All original code used in the study have been deposited at Dryad repository and are publicly accessible as of the date of publication. The DOI is provided in the key resources table. • Any additional information required to reanalyze the data reported in this paper is available from the lead contact upon reasonable request.
