# Supplementary material for: Prosociality in a despotic society
Source: iScience. 2023 Apr 8;26(5):106587. doi: 10.1016/j.isci.2023.106587 (PMC10134446; doi:10.1016/j.isci.2023.106587)
Supplement: Document S1. Figures S1–S4 and Tables S1–S13 [file mmc1.pdf]

iScience, Volume *26*

Supplemental information

Prosociality in a despotic society

Debottam Bhattacharjee, Eythan Cousin, Lena S. Pflüger, and Jorg J.M. Massen

Table S1. List and details of participating individuals, related to STAR Methods. Individuals who participated in the study voluntarily, out of the ~170 individuals present in the population.

Individuals	Age (yo)	Age group	Year of birth	Sex	Matriline rank
Andy	1	Juvenile	2021	♂	10
Cinderella	2	Juvenile	2022	♀	7
Clara	5	Adult	2017	♀	10
Dornroschen	2	Juvenile	2020	♀	5
Frau Holle	2	Juvenile	2020	♀	4
Frida	1	Juvenile	2021	♀	1
Fuji	3	Juvenile	2019	♂	5
Goldmarie	2	Juvenile	2020	♀	7
Herta	10	Adult	2012	♀	5
Iris **(α)**	21	Adult	2001	♀	1
Janis	8	Adult	2014	♀	7
Jessy **(β)**	12	Adult	2010	♀	1
Kate	11	Adult	2011	♀	1
Kiki	1	Juvenile	2021	♀	2
Krato	9	Adult	2013	♀	5
Lisa	11	Adult	2001	♀	5
Marie	5	Adult	2017	♀	4
Pauli	22	Adult	2000	♂	2
Pippi	6	Adult	2016	♀	2
Salvador	1	Juvenile	2021	♂	4
Sandra	11	Adult	2011	♀	4
Spooky **(α)**	10	Adult	2012	♂	3
Uschi	10	Adult	2012	♀	7
Wicky **(β)**	10	Adult	2012	♂	6
Zarah	7	Adult	2015	♀	7

The matriline number refers to the matriline rank and dominance already assessed for this group [1]. All individuals from the same matriline are related to a certain degree and thus are kin relatives. The alpha and beta (males and females) of the group are indicated. Individuals older than four years old were considered adults, and individuals younger than four and higher than or equal to one were juveniles [2].

Figure S1. Food distribution during phase 2 of the group service paradigm, related to STAR Methods. A total of 250 food rewards were placed over two sessions, out of which 177 rewards were obtained by the focal individuals (non-focal individuals were not included in the analysis, i.e., only the 25 participating monkeys were considered).

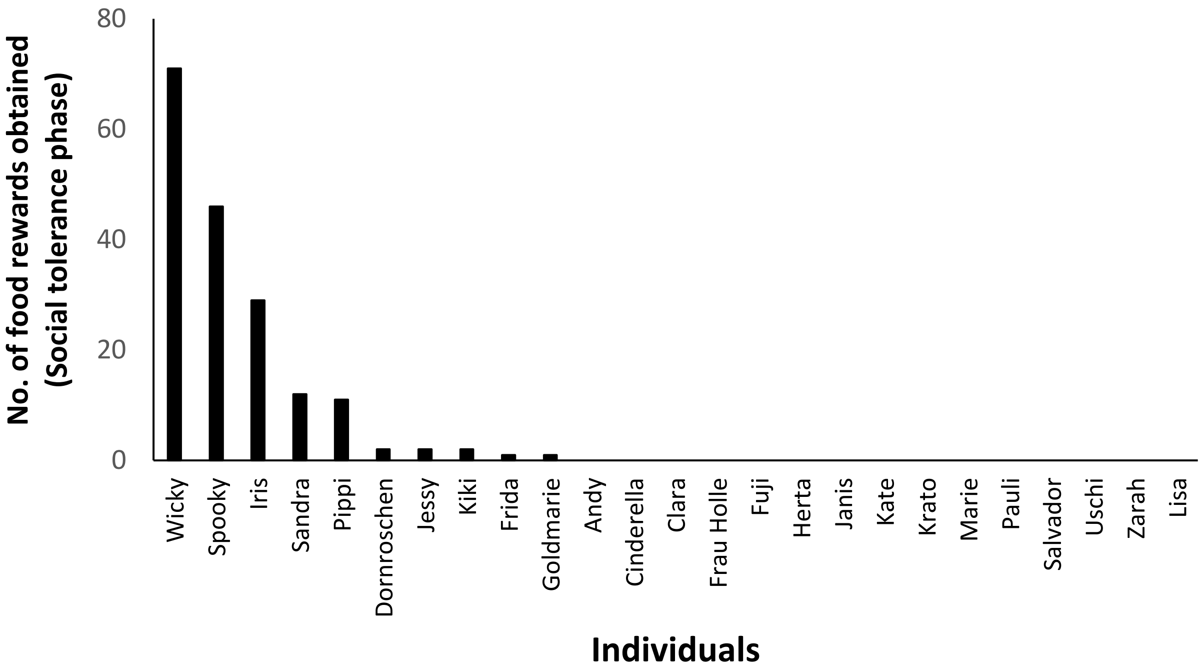

Table S2. Overview of individuals pressing the handle in sessions 4 and 5 of the group service paradigm, related to STAR Methods, and Figure 2.

	Sessions 4 & 5		
**Individuals**	**Test**	**Empty control**	**Blocked control**
Andy	1	0	7
Cinderella	15	23	0***
Clara	1	3	3
Dornroschen	85	4***	3***
Frau Holle	9	0**	5
Frida	22	3***	13
Fuji	17	0***	1***
Goldmarie	2	5	0
Herta	5	4	5
Iris	0	2	0
Janis	1	7	4
Jessy	10	0**	3
Kate	8	3	5
Kiki	4	0	5
Krato	4	5	6
Lisa	3	0	2
Marie	19	20	4**
Pauli	4	0	2
Pippi	0	3	0
Salvador	1	3	5
Sandra	9	0**	8
Spooky	6	1	2
Uschi	0	0	0
Wicky	20	5**	2***
Zarah	7	0*	2

Names in italics represent individuals with prosocial preferences (*** $p < 0.001$, ** $p < 0.01$, * $p < 0.05$, Fisher's exact tests).

Figure S2. Latency of pressing the handle in different conditions, related to STAR Methods. Individuals were faster at pressing the handle in the test (18.32 ± 22.31 sec) than in empty (45.37 ± 29.18 sec) and blocked (44.52 ± 32.18 sec) control conditions (Linear effect model). Half-violin plots indicate the distribution, while solid dots indicate the raw values. The boxes illustrate the interquartile range, horizontal bars inside the boxes indicate median values and whiskers indicate the range of the data.

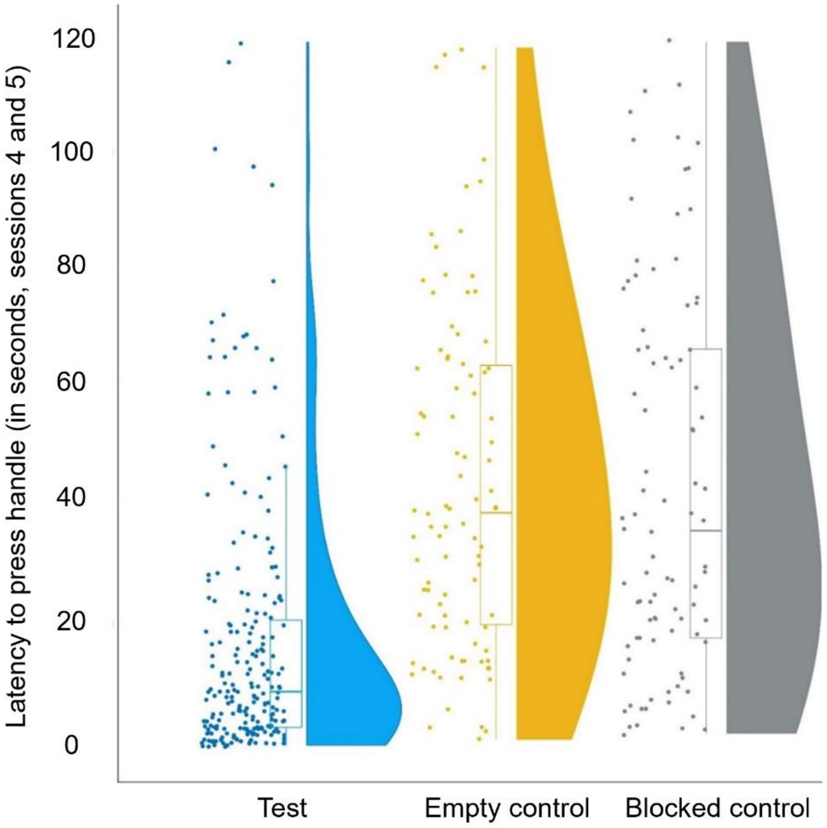

Figure S3. Overview of food provision by individuals across all sessions in the test condition, related to Figure 3. S1-S5: regular sessions, while S6 and S7 indicate re-test sessions. The individuals with prosocial preferences provisioned food at the following rates - S1: 31%; S2: 39%; S3: 43%; S4: 67%; S5: 70%; S6: 65%; S7: 58%.

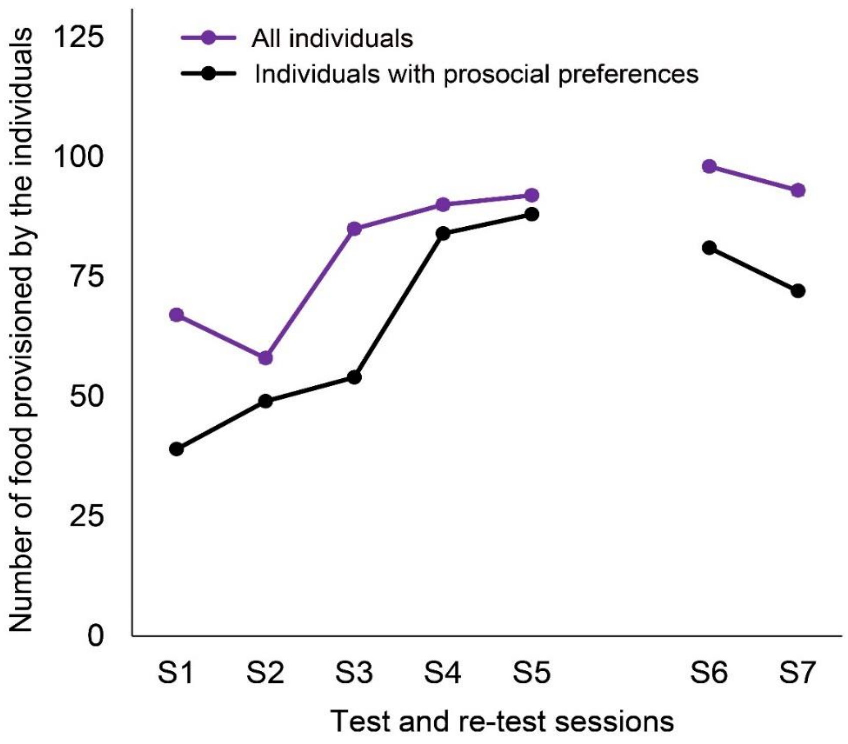

Figure S4. Different sections of the Affenberg Landskron, related to STAR Methods (Photos by Roy Hammer).

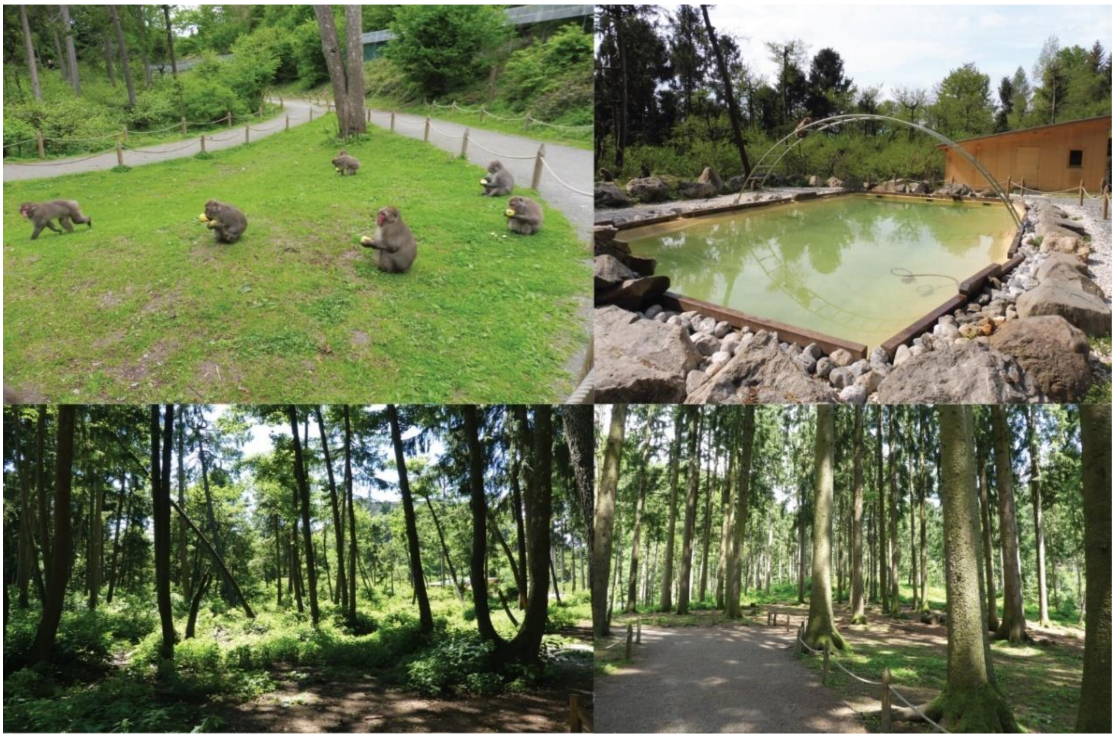

Table S3. Generalized linear mixed model (GLMM) with a Poisson distribution investigating the number of presses (sessions 4 and 5 combined) across experimental conditions, related to Figure 2.

Full model: Number of presses ~ Condition (test/empty control/blocked control) + (1|id), family = "Poisson" (*AIC* = 545.2)

Null model: Number of presses ~ 1 + (1|id), family = "Poisson" (*AIC* = 657.2)

Full model output:

	Estimate	Standard error	z value	p-value
Intercept	1.9231	0.1928	9.972	<2e-16 ***
Condition (empty control)	-1.0225	0.1222	-8.365	<2e-16 ***
Condition (blocked control)	-1.0675	0.1243	-8.589	<2e-16 ***

[Significance code (p-value): 0 '***']

Null vs. Full model comparison: Likelihood ratio test: : $\chi^2 = 115.96$, $p < 0.001$

Table S4. Negative binomial GLMM investigating the number of presses in test (sessions 4 and 5 combined) and re-test (sessions 6 and 7 combined) sessions, related to Figure 3.

Full model: Number of presses ~ session (test/re-test) + (1|id) (*AIC* = 314.1)

Null model: Number of presses ~ 1 + (1|id) (*AIC* = 313.5)

Full model output:

	Estimate	Standard error	z value	p-value
Intercept	1.3339	0.3095	4.309	1.64e-05 ***
Session (test)	0.2943	0.2522	1.167	0.243

[Significance code (p-value): 0 '***']

Table S5. Negative binomial GLMM investigating the number of presses in empty control (sessions 4 and 5 combined) and re-empty control (sessions 6 and 7 combined) sessions, related to Figure 3.

Full model: Number of presses ~ session (empty control/re-empty control) + (1|id) (*AIC* = 228.8)

Null model: Number of presses ~ 1 + (1|id) (*AIC* = 227.1)

Full model output:

	Estimate	Standard error	z value	p-value
Intercept	0.7647	0.4418	1.731	0.0835
Session (re-empty control)	0.2321	0.4543	0.511	0.6095

Table S6. Negative binomial GLMM investigating the number of presses across test sessions (sessions 1-5), related to Figure 3.

Full model: Number of presses ~ session (one/two/three/four/five) + (1|id) (*AIC* = 609.4)

Null model: Number of presses ~ 1 + (1|id) (*AIC* = 615.1)

Full model output:

	Estimate	Standard error	z value	p-value
Intercept	0.9876	0.3589	2.752	0.00593 **
Session (two)	-0.2816	0.3849	-0.732	0.46427
Session (three)	-0.1300	0.3861	-0.337	0.73642
Session (four)	-0.1619	0.4055	-0.399	0.68964
Session (five)	0.2603	0.3968	0.656	0.51191

[Significance code (p-value): 0.001 '***']

Table S7. Linear model investigating the latencies of pressing the handle across experimental conditions and sessions (sessions 4 and 5), related to STAR Methods.

Initial model: Latency to press ~ condition (test/empty control/blocked control) * Session (four/five).

Multicollinearity detected: VIF = 5.92, therefore, the interaction term dropped in the next model -

Full model: Latency to press ~ condition (test/empty control/blocked control) + Session (four/five).

Null model: Latency to press ~ 1

Full model output:

	Estimate	Standard error	z value	p-value
Intercept	1.00607	0.03852	26.119	<2e-16 ***
Condition (empty control)	0.55342	0.05775	9.584	<2e-16 ***
Condition (blocked control)	0.52134	0.05763	9.047	<2e-16 ***
Session (four)	-0.04785	0.04636	-1.032	0.303

[Significance code (p-value): 0 '***']

Null vs. Full model comparison:

Likelihood ratio test: : $\chi^2 = 118.43$, $p < 0.001$

Table S8. Negative binomial GLMM investigating the number of food provisions across sessions in the test condition (Sessions 1-5), related to Figure 2 and Figure 3.

Full model: Number of food provisions ~ session (one/two/three/four/five) + (1|id) (*AIC* = 243.8)

Null model: Number of food provisions ~ 1 + (1|id) (*AIC* = 242)

Full model output:

	Estimate	Standard error	z value	p-value
Intercept	0.1535	0.7751	0.198	0.8431
Session (two)	0.3083	0.7617	0.405	0.6856
Session (three)	0.7674	0.8420	0.911	0.3621
Session (four)	1.5258	0.8889	1.716	0.0861
Session (five)	2.0338	0.8824	2.305	0.0212 *

[Significance code (p-value): 0.05 '*']

Null vs. Full model comparison:

Likelihood ratio test: : $\chi^2 = 6.234$, $p = 0.18$

Table S9. Number of food provided and received during the S4 and S5 phases of the test condition of the group service paradigm, related to Figure 2.

Individuals	Provided	Received
Andy	0	0
Cinderella	11	3
Clara	1	3
Dornroschen	78	9
Frau Holle	9	7
Frida	22	1
Fuji	17	5
Goldmarie	1	3
Herta	0	1
Iris	0	0
Janis	1	5
Jessy	0	28
Kate	2	6
Kiki	2	25
Krato	2	10
Lisa	0	41
Marie	15	3
Pauli	1	2
Pippi	0	0
Salvador	0	10
Sandra	7	9
Spooky	0	0
Uschi	0	0
Wicky	11	0
Zarah	2	0

Table S10. Binomial GLMM investigating the likelihood of food provisioning, related to Figure 4.

Initial model: Food provision (yes/no) ~ kinship * dyadic social tolerance + age difference (yes/no) + sex difference (yes/no) + rank difference (yes/no) + (1|actor/receiver)

Model convergence issues, hence, interaction term dropped in the next model –

Full model: Fod provision (yes/no) ~ kinship + dyadic social tolerance + age difference (yes/no) + sex difference (yes/no) + rank difference (yes/no) + (1|actor/receiver) (*AIC* = 107.1)

Null model: Food provision (yes/no) ~ 1 + (1|actor/receiver) (*AIC* = 177)

Full model output:

	Estimate	Standard error	z value	p-value
Intercept	-15.8043	5.97912	-2.643	0.00821 **
Kinship (yes)	3.53438	3.01227	1.173	0.24066
Dyadic social tolerance	1.61025	0.63941	2.518	0.01179 *
Age difference (yes)	0.72333	2.88402	0.251	0.80196
Sex difference (yes)	1.25921	2.95683	0.426	0.67021
Rank difference (high)	-0.06415	3.23779	-0.020	0.98419

[Significance code (p-value): 0.01 '**', 0.05 '*']

Null vs. Full model comparison:

Likelihood ratio test: : $\chi^2 = 79.96$, $p < 0.001$

Table S11. Binomial GLMM investigating the likelihood of food provisioning using a substitute model to the model described in Table S10, related to Figure 4.

Substitute model: Food provision (yes/no) ~ kinship + dyadic social tolerance + age difference (adult to juvenile/juvenile to adult/adult to adult/juvenile to juvenile) + sex difference (female to male/female to female/male to female/male to male) + rank difference (yes/no) + (1|actor/receiver) (*AIC =114.8*)

Model output:

	Estimate	Standard error	z value	p-value
Intercept	-15.6536	5.8669	-2.668	0.00763 **
Kinship (yes)	3.1643	2.9581	1.070	0.28476
Dyadic social tolerance	1.4149	0.5932	2.385	0.01707 *
Age difference (adult-juvenile)	1.3902	5.0004	0.278	0.78100
Age difference (juvenile-adult)	-1.0570	3.5589	-0.297	0.76647
Age difference (juvenile-juvenile)	-1.3342	4.7487	-0.281	0.77874
Sex difference (female-male)	0.4116	3.8503	0.107	0.91487
Sex difference (male-female)	2.0650	3.3549	0.616	0.53821
Sex difference (male-female)	-8.5656	808.69	-0.011	0.99155
Rank difference (high)	1.3471	4.5537	0.296	0.76737

[Significance code (p-value): 0.01 '**', 0.05 '*']

Table S12. Poisson GLMM investigating the magnitude of food provisioning, related to Figure 4.

Initial model with interactions: Number of provisions ~ Kinship * Dyadic social tolerance + Age class + Sex class + Dominance rank difference + (1|actor/receiver), family = "Poisson"

[No interaction was found between the fixed effects of kinship and dyadic social tolerance. Thus, the main effects were investigated]

Full model: Number of provisions ~ Kinship + Dyadic social tolerance + Age class + Sex class + Dominance rank difference + (1|actor/receiver), family = "Poisson" (*AIC = 163.2*)

Best-fitted model: Number of provisions ~ Kinship + Dyadic social tolerance + Dominance rank difference + (1|actor/receiver), family = "Poisson" (*AIC = 159.8*)

Null model: Number of provisions ~ 1 + (1|actor/receiver), family = "Poisson" (*AIC = 169.3*)

Best-fitted model output:

	**Estimate**	**Std. Error**	**z value**	**Pr(>|z|)**
(Intercept)	0.47656	0.30993	1.538	0.12414
Kinship (yes)	0.93666	0.31386	2.984	0.00284 **
Dyadic Social tolerance	0.06514	0.03159	2.062	0.03921 *
Rank difference (high)	0.44218	0.33832	1.307	0.19121

[Significance codes: p < 0.01‘**’, 0.05 ‘*’]

Null vs. Full model comparison: Likelihood ratio test: $\chi^2 = 15.5$, $p = 0.001$

Table S13. Poisson GLMM investigating the magnitude of food provisioning using a substitute model to the model described in Table S12, related to Figure 4.

Substitute model: Number of provisions ~ kinship + dyadic social tolerance + age difference (adult to juvenile/juvenile to adult/adult to adult/juvenile to juvenile) + sex difference (female to male/female to female/male to female/male to male) + rank difference (yes/no) + (1|actor/receiver) (*AIC =170*)

Best-fitted substitute model: Number of provisions ~ kinship + dyadic social tolerance + rank difference (yes/no) + (1|actor/receiver) (*AIC =163.7*)

Best-fitted substitute model output:

	Estimate	Standard error	z value	p-value
Intercept	0.43219	0.31805	1.359	0.17419
Kinship (yes)	1.00305	0.32221	3.113	0.00185 **
Dyadic Social tolerance	0.07009	0.03264	2.147	0.03179 *
Rank difference (high)	0.37643	0.34640	1.087	0.27717

[Significance code (p-value): 0.01 ‘**’, 0.05 ‘*’]

References

[1] Nakagawa, N., 2010. Intraspecific Differences in Social Structure of the Japanese Macaques: A Revival of Lost Legacy by Updated Knowledge and Perspective. pp. 271–290. https://doi.org/10.1007/978-4-431-53886-8_12

[2] Pflüger, L.S., Pink, K.E., Wallner, B., Radler, C., Dorner, M., Huffman, M.A., 2021. Twenty-three-year demographic history of the Affenberg Japanese macaques (Macaca fuscata), a translocated semi-free-ranging group in southern Austria. Primates 62, 761–776. https://doi.org/10.1007/s10329-021-00928-4
